# The Efficacy of Gabapentin in Patients with Central Post-stroke Pain

**Published:** 2015

**Authors:** Omid Hesami, Kourosh gharagozli, Nahid Beladimoghadam, Farhad Assarzadegan, Behnam Mansouri, Mohammad Sistanizad

**Affiliations:** a *Department of Neurology, Emam Hossein Medical and Educational Center, Shahid Beheshti University of Medical Sciences, Tehran, Iran. *; b *Loghman Medical and Educational Center, Shahid Beheshti University of Medical Sciences, Tehran, Iran.*; c *Department of Clinical Pharmacy, School of Pharmacy, Shahid Beheshti University of Medical Sciences, Tehran, Iran. *; d *Pharmaceutical Care Unit, Emam Hossein Medical and Educational Center, Shahid Beheshti University of Medical Sciences, Tehran, Iran.*

**Keywords:** Gabapentin, Central nervous system, Cerebrovascular accident, Central post stroke pain

## Abstract

Thalamic pain syndrome, a type of central post-stroke pain (CPSP), may develops after a hemorrhagic or ischemic stroke and results in impairment of the thalamus. There is limited experience about gabapentin in treatment of central pains like CPSP.

In a prospective observational study, the intensity of pain was recorded using the Numeric Rating Scale (NRS) at the entrance to the study. Patients eligible for treating with gabapentin, received gabapentin 300 mg twice-daily. The pain intensity was measured at entrance to the study and after one month using NRS. Decrease of 3 points from the initial NRS considered being clinically significant.

From a total of 180 primarily screened patients, 84 (44 men and 40 women) were recruited. There was a significant difference between pre-treatment and post-treatment NRS (5.9 ± 2.51 vs. 4.7 ± 3.01; 95% CI: 0.442-1.962, p = 0.002). Fisher's exact test showed no statistically significant effect of clinical and demographic characteristics of patients on their therapeutic response to gabapentin.

Given the safety, efficacy, well tolerability and lack of interaction with other drugs we suggest gabapentin to be more considered as a first line therapy or as add-on therapy for reducing the pain severity in patients with thalamic syndrome.

## Introduction

Central post-stroke pain (CPSP) can be defined as a central neuropathic pain condition occurring after stroke characterized by pain and sensory abnormalities where other causes of obvious nociceptive, psychogenic, or peripheral pain have been excluded (1, 2). The prevalence and yearly incidence of CPSP have been reported as 7.3% and 8%, respectively (2, 3) and occurs in 8%–14% of patients with stroke (4). CPSP is considered challenging from a clinical management standpoint and existing treatment options do not result in optimal outcomes (5, 6).

Although it is more than a century after the first description of the thalamic syndrome, the evidence for treatment of this pain syndrome is still extremely diminutive. Pain medications often provide some reduction of pain, but not complete relief of pain, for those affected by central pain syndrome. Opioids, tricyclic antidepressants such as amitriptyline or anticonvulsants such as lamotrigine and gabapentin have been used in patients with CPSP (7, 8). Because of potential side effects, long-term use of IV drugs such as lidocaine, propofol and ketamine have been limited; although they might be beneficial for short therapeutic episodes (7). Few pilot studies have reported Kampo medicine (9) and vestibular caloric stimulation (10) as effective treatments in patients refractory to conventional pharmacologic treatments. Despite several studies and reports debate continues about the best strategies for the management of CPSP and there are still too few large-scale comparative studies (6). 

Gabapentin and pregabalin, safe and partially well tolerated antiepileptic agents, have shown efficacy in several neuropathic pain conditions (-). It has been approved as adjunctive therapy in the treatment of partial seizures and for the management of postherpetic neuralgia (15) and currently is also used for modulating the peripheral and central neuropathic pains (16) and relieving withdrawal-related pain due to heroin use (13). 

Despite several studies of gabapentin in the area of neuropathic pain, far too little attention has been paid to its role in CPSP. Serpel *et al.* in a randomized, placebo-controlled trial of 305 patients with chronic pain, 9 of whom had CPSP, evaluated the efficacy and safety of gabapentin in the treatment of neuropathic pain. But the effect of gabapentin was not significant in patients suffering CPSP (17). Usefulness of gabapentin, however, has been reported in a 45-yr old man with CPSP who was refractory to phenytoin, carbamazepine, and sodium valproate(8).

The aim of this study was to determine the efficacy of gabapentin in the management of patients suffering CPSP with regard to reduction in numerical rating scale for pain scores (NRS).

## Experimental

*Study design and setting*

This was a prospective observational study conducted in multi centers at Tehran, Iran. Participating centers were two outpatient clinics from two different educational hospitals and three private neurology clinics in Tehran, Iran. Approval of this study was conducted by the institutional review board of all of the participating centers. All patients were given a written informed consent before entering the study, and the study was performed in accordance with the declaration of Helsinki.

All patients with stroke in the thalamic area admitted to each center between January 7, 2010 and June 30, 2011 were evaluated. Patients documented as being diagnosed with CPSP and who found to be eligible for treating with gabapentin as their standard therapy, entered to the study and demographic information (age, gender), stroke type, NRS on day of diagnosis, and NRS 30-day after treatment initiation were documented.

Management of the patients was based on EFNS (European Federation of Neurological Societies) guidelines on the pharmacological treatment of neuropathic pain (6). According to each patient’s condition and comorbidities and past medical history, appropriate analgesic drug, which was one anticonvulsant or tricyclic antidepressant, was given as their standard therapy. 

If a patient with an initial treatment other than gabapentin had no clinical response to the drug, a proper response was defined as at least three points decline in NRS, the therapeutic course was discontinued. NRS was recorded again and gabapentin was started for the patient. Finally, one month after initiation of gabapentin, the NRS of the patients were documented. 

*Study population*

Patients were qualified for the study on the basis of these inclusion criteria: (I) positive history of hemorrhagic or ischemic stroke in the thalamic area proved by computed tomography or magnetic resonance imaging of the brain; (II) presence of signs and symptoms in accordance with a decrease in pain and heat sensations in the affected part of the body; (III) presence of spontaneous or stimulated pain in the affected side (contralateral to the thalamic involvement) which could be smaller or the same in size as the sensory impairment area; and (IV) a pain onset after a thalamic stroke. 

Exclusion criteria comprise lack of imaging findings compatible with stroke and other imaging abnormalities including tumors in the thalamus area; absence of sensory impairment in the affected side of the body; therapeutic response to other treatments of thalamic syndrome; and previous hypersensitivity to gabapentin. Women were excluded if they were pregnant or lactating during the study. Eligible patients received gabapentin 300 mg twice daily for one month.

*Outcome measures*

The primary outcome in this study was the change in the NRS from the baseline through the fourth week of treatment and the secondary outcome was the proportion of patients with a clinically significant change in NRS (three-point decrease). 

*Statistical analysis*

Comparison was done using the paired sample t-test. Two-sided 95% confidence interval (CI) for the difference in response rates was calculated. A p-value of < 0.05 was needed for a result to be considered statistically significant. In the second step of our analysis, patients with a proper response and those who did not response to the suggested treatment were compared with regard to their baseline characteristics to find any probable correlation. To perform this comparison, unpaired t-test was used for interval variables and Chi-square or Fisher's exact test was used for nominal data. 

## Results

*Study patients*

**Figure 1:**
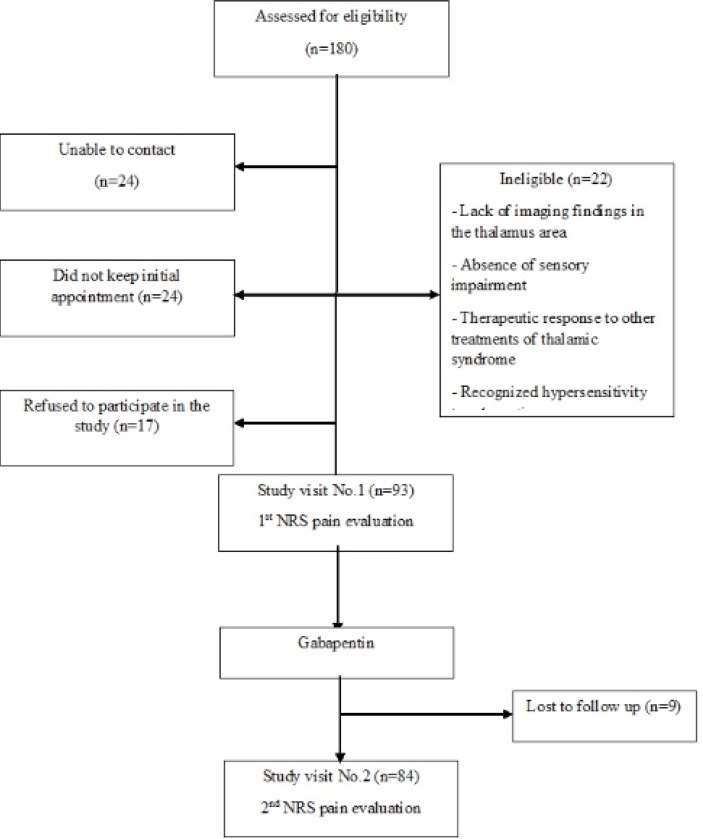
Flowchart of the study; NRS, Numeric Rating Scale

Of all 180 primarily screened patients, 93 patients entered to the study of whom 9 did not complete the study during the follow up period, and 84 patients successfully completed the study (Figure 1).

Among patients 44 (52.4%) men and 40 (47.6%) women completed the study. Type of underlying stroke that caused thalamic syndrome was ischemic in 76 (90.5%) patients and hemorrhagic in 8 (9.5%) patients. Concurrent hyperlipidemia, hypertension and diabetes mellitus as coexistent diseases were present in 9 (10.7%), 54 (64.3%) and 17 (20.2%) of the included patients, respectively. NRS score change from day one to day thirty was significantly different between pre-treatment (6.7 ± 2.51) and post-treatment (3.4 ± 3.01), (CI 0.442-1.962, p = 0.002).

*Comparing patients with adequate and inadequate responses*

Our results found that 50 patients (59.5%) showed a clinically significant improvement according to NRS scores (3 points decline in post-treatment NRS). In the second step of our analysis we tried to find probable effects of patients` demographics and clinical characteristics on their response to the suggested regimen.

Our findings revealed that distribution of gender, age category, previous history of diabetes mellitus; hypertension; hyperlipidemia, and stroke-type were not statistically different between the responders and non-responders. These findings are presented in Table 1.

**Table 1:** Demographic and clinical characteristics of patients and their effect on therapeutic response with gabapentin

**Characteristics**	**Responders**	**Non-responders**	**Total**	**p-** **value**
**Gender**				
Male	25(29.7%)	19(22.6%)	44(52.4%)	N.S
Female	25(29.7%)	15(17.8%)	40(47.6%)	N.S
**Age groups**				N.S
<40	3(3.5%)	1(1.1%)	4(4.8%)	
41-50	6(7.1%)	6(7.1%)	12(14.3%)	
51-60	15(17.8%)	11(13%)	26(31%)	
61-70	17(20.2%)	12(14.2%)	29(34.5%)	
>70	9(10.7%)	4(4.7%)	13(15.5%)	
**Underlying disease**				
Hyperlipidemia	6(7.1%)	3(3.5%)	9(10.7%)	N.S
Hypertension	36(42.8%)	18(21.4%)	54(64.3%)	N.S
Diabetes mellitus	11(13%)	6(7.1%)	17(20.2%)	N.S
**Type of stroke**				
Hemorrhagic	7(8.3%)	1(1.1%)	8(9.5%)	N.S
Ischemic	43(51.1%)	33(39.2%)	76(90.5%)	N.S

## Discussion

In this multicenter study we showed that in more than half of patients with central post stroke pain, the NRS pain score was reduced after a month of therapeutic course with gabapentin.

For patients with thalamic syndrome due to severity and chronicity, like other types of chronic pain, reduction of pain intensity, instead of pain elimination, should be considered as the goal of the treatment strategy. Hence, in the present study and in order to perform a better evaluation of pain intensity we used NRS. By NRS, patients will be asked about their pain and this individualized scoring can help physician make a better assessment.

Central pain syndrome is a neurological condition that originates from damage to the central nervous system (CNS) or may happen due to impairment of the function of CNS. Many conditions such as stroke, tumors of CNS and spinal cord, multiple sclerosis, epilepsy, brain or spinal cord trauma or can be served as an underlying process and induce this pain syndrome.

The probable pathophysiology of thalamic syndrome includes a post-stroke lesion of thalamus that results in toxic, anatomical and inflammatory changes which simultaneously ruins the inhibitory mechanisms of the sensory thalamus and its related neuronal pathways and also can destruct the afforestation of the sensory pathways and eventually results in neuronal hyper excitability above the level of differentiation (-). This hyperactivity can result in central sensitization, which in turn leads to chronic pain (21).

The diversity and convolution of damages affected multiple parts of the spinothalamocortical pathway, indicates the intricate pathophysiology of this pain and clarifies the challenge of its effective treatment.

Gabapentin (Neurontin), 1–(aminomethyl) cyclohexane–acetic acid, is an antiseizure drug primarily approved for treatment of partial seizures with an undocumented mechanism of action, however recent studies have reported that probably gabapentin elevates gamma-Aminobutyric acid (GABA) levels in the different parts of the brain including the thalamus as well as inducing GABA release from glial cells. Although, the action sites of gabapentin for pain reduction has not been well clarified but both animal (-) and human (25) studies have shown both spinal and supraspinal sites of action.

Several reports have been demonstrated gabapentin as a first line treatment (-) or as an add-on therapy in combination with other drugs (29) in patients suffering from peripheral neuropathic pains such as post-herpetic neuralgia, sciatic type pain and diabetic neuralgia. This is while specific research on gabapentin to treat central pains is limited to case series and low quality clinical trials. So far, gabapentin has been shown to be efficacious in relieving the spontaneous and paroxysmal pain caused by peripheral and central lesions (30). 

In a case report published by Chen *et al*. (8), gabapentin was suggested as an effective and well-tolerated drug for a patient with central post-stroke pain syndrome who failed to respond to a variety of oral analgesics. 

Similarly, in our study all of the patients tolerated the drug well and no one discontinued the therapeutic course because of the adverse events of the drug.

Previously, gabapentin has been shown as an effective second line medication to decrease the pain intensity in cases who failed to achieve a proper response after standard treatment with first line agents like lamotrigine and amitriptyline (4).

Amitriptyline, from the antidepressants group, was among the first drugs proven to be effective in central post stroke pain syndrome in a double-blind placebo-controlled study (31). Lamotrigine, as an anti-epileptic medication with non-NMDA anti-glutamatergic activity, has been proposed as an alternative to tricyclic antidepressants in the treatment of central post stroke pain. Analgesic abilities of lamotrigine in reduction of both the spontaneous and evoked components of central pains have been evaluated in a case series and its efficacy has been proven (32).

Due to possible dose dependent adverse effects of gabapentin, this study tried low doses of this drug. In our study, the proposed daily dosage of 600 mg yielded a significant clinical improvement with no adverse effects. Serpell and colleagues have evaluated clinical efficacy of higher doses of gabapentin titrated to 2400 mg/day in 9 patients with central post stroke pain over an eight weeks period (17). Clinical outcomes of that study are comparable to the findings of the present study but we achieved our results with a very lower dosage. Safety and efficacy of gabapentin with a limited dose makes this agent a superior therapeutic option in comparison with conventional analgesics.

Usual analgesics, including opioids, are generally ineffective in treatment of central post stroke pain and due to their possible adverse events; it is generally recommended that they should be avoided. In a study published by Attal *et al.* (33), there was no significant difference between the effects of morphine and placebo. In that study, only a minority of patients got benefit from long-term treatment with opioids. Moreover, the use of opioids as oral agents in pain reduction in patients with central and peripheral neuropathic pain has been trialed in a study by Rowbotham *et al.* (34). The study demonstrated that patients with central pain, compared with those with peripheral neuropathic pain, were less likely to obtain benefits. 

We also attempted to determine the probable influence of baseline characteristics on the therapeutic response, and found that baseline type of stroke, age, gender, positive history for hyperlipidemia; hypertension and diabetes mellitus did not have a statistically significant effect on response to treatment. Further baseline characteristics analyses are needed to help identify probable gabapentin response factors.

In conclusion, the findings of this study show gabapentin as an effective and well tolerable treatment for patients with thalamic syndrome. There is a paucity of data toward the efficacy of this drug in pain relief in patients with thalamic syndrome, so we suggest more clinical trials to be conducted to evaluate various aspects of using gabapentin in treating patients with thalamic syndrome. Given the safety and lack of interaction with other drugs we suggest gabapentin to be considered as a first line therapy or as add-on therapy for reducing the pain severity in patients with thalamic syndrome. A randomized controlled trial with a larger sample size may attenuate methodological weaknesses of the present trial and open new avenues for a successful usage of gabapentin in these patients.
